# Supplementary material for: Bi-frequency operation in a membrane external-cavity surface-emitting laser
Source: PLoS One. 2023 Jul 27;18(7):e0289223. doi: 10.1371/journal.pone.0289223 (PMC10374101; doi:10.1371/journal.pone.0289223)
Supplement: Attachment — Submitted filename: Review PONE-D-23-16239.pdf [file pone.0289223.s001.pdf]

Review of the paper “Bi-frequency operation in a membrane external-cavity surface-emitting laser” by J. Daykin, et al., submitted to *PLOS ONE*.

In this paper, the authors demonstrated an optically pumped bi-frequency MECSEL. One of the cavity mirrors is laser ablated to introduce a transverse-mode-dependent loss, making it possible for two transverse modes to lase simultaneously with similar output power.

The manuscript introduces details on the laser structure including the ablated mirror, cavity, gain medium, and pump laser. Operations under various pump power and different ablated masks are experimentally studied. The results show promising bi-frequency operations with tuning capabilities. I would like to recommend the manuscript be published after addressing a few questions below:

1. The acronym “VECSEL” first appears in line 7, while the full term “Vertical External-Cavity Surface-Emitting Laser” appears later in line 14. Please make sure the acronym is defined at the first appearance.
2. In line 61, the masks are described as “with ablated central regions”. However, the actual masks seem to have unablated central regions. Can the authors clarify this?
3. In line 132, the authors mentioned Fig. 5 has a 300-mW increment of pump power. The pump power range in Fig. 5 plots is about 2.5 ~ 4.0 W, so there should be only 5 or 6 discrete pump power values. However, some plots in Fig. 5 show more than 6 different spectrum sections across the pump power range, so it seems the increment is less than 300 mW. Can the authors clarify this?
4. In Fig. 5, is the colormap in log scale or linear scale? Some plots seem to have a higher noise floor at some pump power values (for example, the 50 um mask plot at about 2.5 W pump power). What is the reason for this?
